# Poor Performance of Large Language Models Based on the Diabetes and Endocrinology Specialty Certificate Examination of the United Kingdom

**DOI:** 10.7759/cureus.93960

**Published:** 2025-10-06

**Authors:** Ka Siu Fan, Jeffrey Gan, Isabelle X Zou, Maja Kaladjiska, Monique B Inguanez, Gillian L Garden

**Affiliations:** 1 Faculty of Health and Medical Science, University of Surrey, Guildford, GBR; 2 Centre for Endocrinology and Diabetes Research, Royal Surrey NHS Foundation Trust, Guildford, GBR; 3 Medical School, Imperial College London, London, GBR; 4 Department of General Medicine, Royal Berkshire Hospital, Reading, GBR; 5 Department of Statistics and Operations Research, University of Malta, Msida, MLT

**Keywords:** artificial intelligence (ai), diabetes and endocrinology, higher education and ai, large language model(llm), medical education

## Abstract

Introduction

The medical knowledge of large language models (LLMs) has been tested using several postgraduate medical examinations. However, it is rarely examined in diabetes and endocrinology. This study aimed to evaluate the performance of LLMs in answering multiple-choice questions using the Diabetes and Endocrinology Speciality Certificate Examination (SCE) of the United Kingdom.

Methods

The official diabetes and endocrinology SCE sample questions were used to assess the seven freely accessible and subscription-based commercial LLMs: ChatGPT-o1 Preview (OpenAI, USA), ChatGPT-4o (OpenAI, USA), Gemini (Google, USA), Claude-3.5 Sonnet (Anthropic, USA), Copilot (Microsoft, USA), Perplexity AI (Perplexity, USA), and Meta AI (Meta, USA). The accuracy of LLMs was calculated by comparing outputs against sample answers. Literacy metrics, including Flesch Reading Ease (FRES) and Flesch Kincaid Grade Level (FKGL), were calculated for each response. 83 questions, three of which included photographs, were entered into the LLMs without employing any prompt engineering techniques.

Results

A total of 581 responses were generated and captured between August and October 2024. Performance differed significantly between models, with ChatGPT-o1 Preview achieving the highest accuracy (73%). None of the other LLMs achieved the historical pass mark of 65%, with Gemini achieving the lowest accuracy of 33%. Readability metrics also differed significantly between LLMs (p=0.004). LLMs performed better for questions without reference ranges (p<0.001).

Conclusions

The performance of LLMs was generally inadequate in the diabetes and endocrinology examination. Of those tested, ChatGPT-o1 Preview achieved the highest score and is likely the most useful model to aid medical education. This may be due to it being an advanced reasoning model with a greater ability to solve complex problems. Nonetheless, continued research is needed to keep pace with the advances in LLMs.

## Introduction

Increasing interest has been shown in the implementation of Artificial Intelligence (AI) into healthcare practice, from supporting patient-level care to population health decision-making [1]. Large Language Models (LLMs) are an increasingly popular form of AI that analyzes large amounts of data to perform natural language processing tasks, including the generation of conversational text. Multiple LLMs, such as ChatGPT and Google Bard, have been shown to pass various postgraduate medical examinations, including the United States Medical Licensing Examination (USMLE) and the intercollegiate Membership of the Royal College of Surgeons (MRCS) [2-5]. Of note, some LLMs allowing image input have successfully answered examination questions containing images in the Speciality Certificate Examination (SCE) in Dermatology [6]. ChatGPT and Bard have previously been tested on endocrine and diabetes-related multiple-choice questions (MCQs) taken from textbooks and university examinations - both LLMs failed to achieve the passing score set at 60% [7]. The use of LLMs in medical education is also being explored, including personalized education of both clinicians and patients [1,8].

The SCE in Endocrinology and Diabetes is a postgraduate examination undertaken as part of the Certificate of Completion of Training in Endocrinology and Diabetes Mellitus in the UK. It consists of two written papers, each containing 100 MCQs covering knowledge of diabetology and endocrinology. Each question includes five multiple-choice options, with one being the ‘most correct’ [9]. SCE questions are designed to apply core knowledge to realistic clinical scenarios, beyond factual recall. As of 2023, the pass mark was set at 65.5%, or 131 out of the total 200 questions. The pass rate for trainees for the 2023 examination was 48.5% for UK trainees and 28.6% for all candidates. The Royal College of Physicians of the United Kingdom (MRCP UK) have released sample questions in a mock examination paper to help candidates prepare.

With previous studies showing poor performance from previous versions of ChatGPT and Bard in questions on diabetes and endocrinology, this study aimed to provide a comprehensive assessment of all the major commercially available LLMs. This study aimed to evaluate the performance of each LLM using the validated questions of the diabetes and endocrinology SCE of the MRCP UK, in order to see how useful these AI tools could be for doctors in training.

## Materials and methods

Study design

This is a cross-sectional study designed to assess the performance of commercially available LLMs. Sample diabetes and endocrinology MCQs and their corresponding official answers were extracted from the MRCP UK website. A total of 83 sample questions, three of which contained images, were available. All of the sample examination questions were considered an accurate representation of the actual examination for this study, though the relative difficulty of these questions was not stated [10].

Seven LLMs were evaluated in this study: ChatGPT-o1 Preview (OpenAI, USA), ChatGPT-4o (OpenAI, USA), Gemini (Google, USA), Claude-3.5 Sonnet (Anthropic, USA), Copilot (Microsoft, USA), Perplexity AI (Perplexity, USA), and Meta AI (Meta, USA). These LLMs were selected for their availability and free usage (with some restrictions). ChatGPT-o1 Preview was included as one of the newest models, which required a paid subscription. Together, the models selected were considered representative of most users of LLMs.

Each LLM was provided with the prompt “Please select the most appropriate option for the following questions:” before the questions were provided in subsequent prompts. For each prompt, an examination question and its five multiple-choice options were given. Images included in questions were uploaded in their original resolution with the corresponding question and multiple-choice options. There was no image input function for ChatGPT-o1 Preview and Meta AI at the time. Questions with images were included in the final analysis as the question stems still had sufficient clinical information for reasoning. The response from each prompt was recorded, and each suggested answer from the LLM was compared against the official answers. The pass mark was 65% based on historical cut-off marks [9].

**Figure 1:**
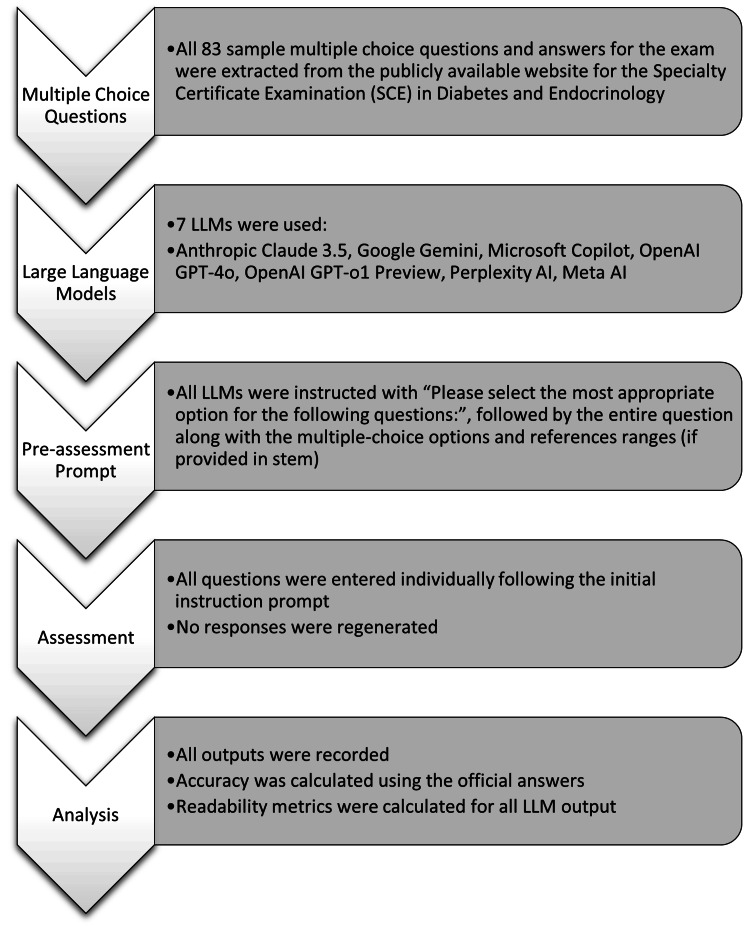
Study workflow ChatGPT-o1 Preview (OpenAI, USA), ChatGPT-4o (OpenAI, USA), Gemini (Google, USA), Claude-3.5 Sonnet (Anthropic, USA), Copilot (Microsoft, USA), Perplexity AI (Perplexity, USA), and Meta AI (Meta, USA). LLM: large language model

To evaluate response readability, the word count, Flesch Reading Ease Score (FRES), and Flesch Kincaid Grade Level (FKGL) were calculated. These metrics provide a widely accepted quantitative assessment of the readability and complexity of the outputted text [11-13]. These are calculated via the number of words, sentences, and syllables, where higher FRES scores indicate less complicated text. FKGL indicates the education level required to understand the text, with FKGL of 12 being equivalent to the reading level of a secondary school graduate in native English-speaking countries. The study workflow is illustrated in Figure 1.

Data analysis

Data was analyzed within Excel (Microsoft, USA) and SPSS v25 (IBM, USA). Shapiro-Wilk normality testing demonstrated that the quantitative variables did not follow a normal distribution. Chi-squared test, Mann-Whitney U test, and Kruskal-Wallis tests were used for analysis. Performance and readability metrics were evaluated for different LLMs and question themes. Questions were also categorized by whether they were focused on diabetes or endocrinology. Statistical significance was set at less than 0.05.

## Results

LLM responses were generated and collected between August and October 2024. A total of 581 responses were recorded: none required additional prompting. All 83 questions were included and were grouped into the following themes: management (n=37), diagnosis/investigation (n=24), and physiology/anatomy (n=21).

**Figure 2:**
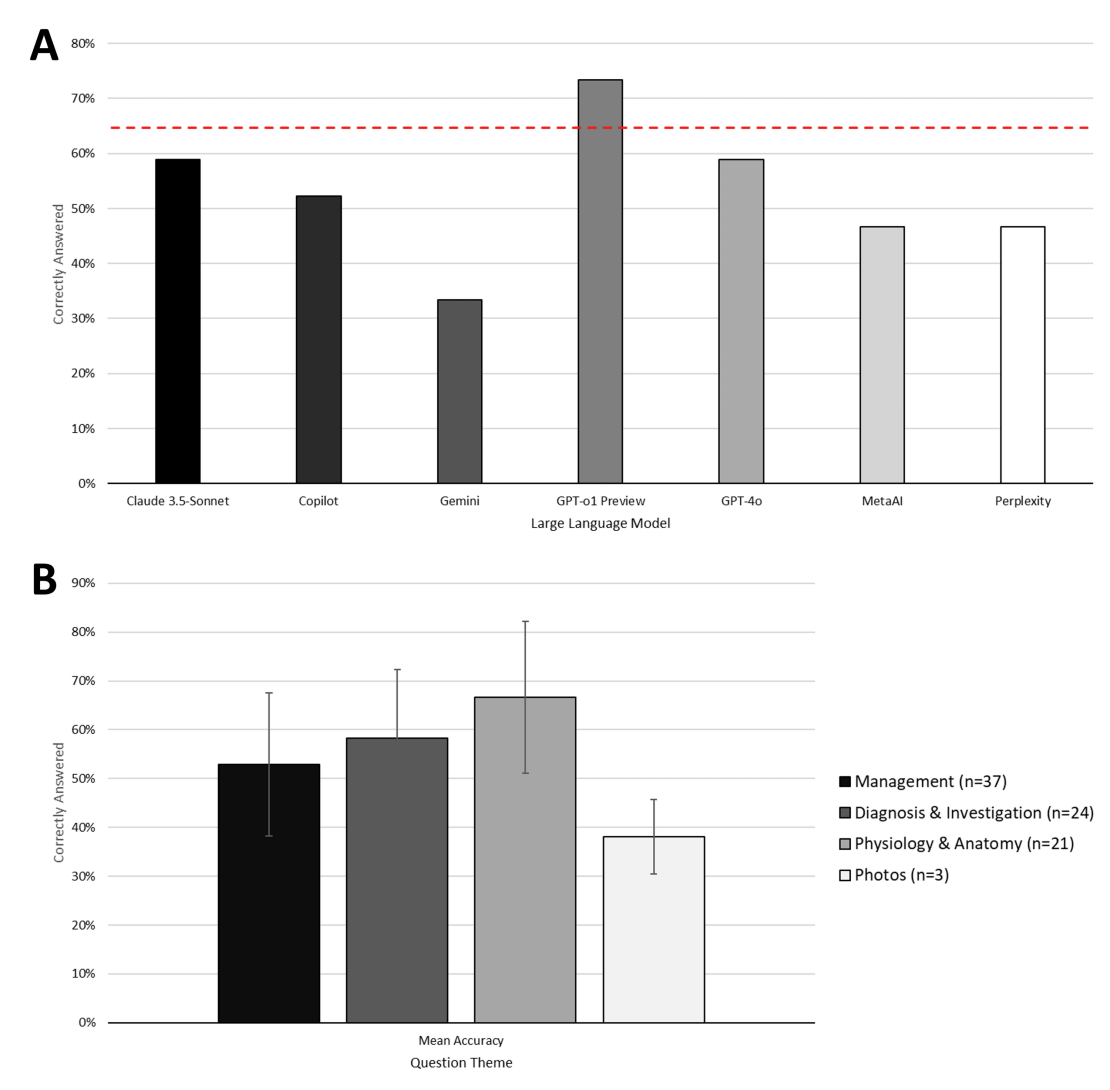
Overview of performance by large language model (A) with red dotted line representing the historical pass mark of ~65%; and performance by question theme (B) ChatGPT-o1 Preview (OpenAI, USA), ChatGPT-4o (OpenAI, USA), Gemini (Google, USA), Claude-3.5 Sonnet (Anthropic, USA), Copilot (Microsoft, USA), Perplexity AI (Perplexity, USA), and Meta AI (Meta, USA).

ChatGPT-o1 Preview achieved the highest score (73%), followed by ChatGPT-4o (59%) and Claude-3.5 Sonnet (59%). Gemini had the lowest performance of 33%. Of all the LLMs, only ChatGPT-o1 Preview achieved the historic pass mark of 65% (Figure 2). 

**Figure 3:**
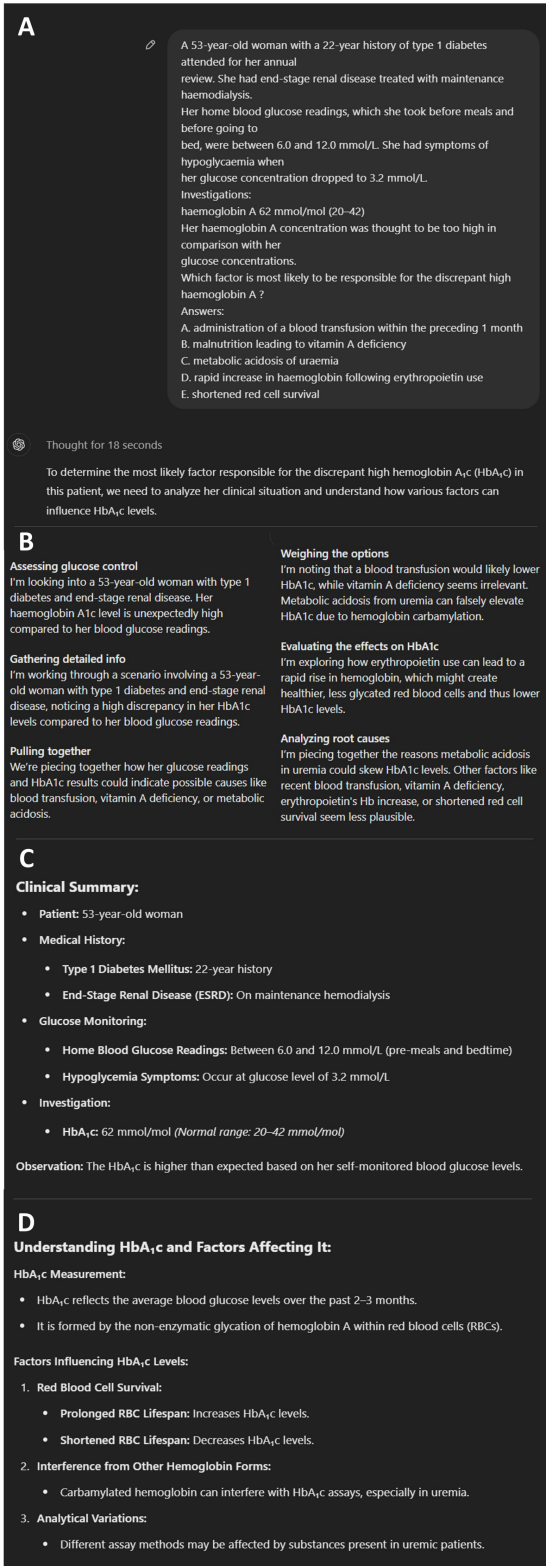
Demonstration of ChatGPT-o1 Preview response and reasoning for an example multiple-choice question on diabetes Figure 3A illustrates the raw input of the question into the interface. Figure 3B shows the expanded reasoning steps taken by the model, which may be accessed by the ‘thought for X seconds’ button. Figure 3C shows the clinical summary of the salient facts within the inputted scenario/prompt. Figure 3D shows the interpretation of the key concept being tested and its relevance to the scenario. Screenshot taken from a ChatGPT chat transcript (ChatGPT; OpenAI, San Francisco, CA, USA).

**Figure 4:**
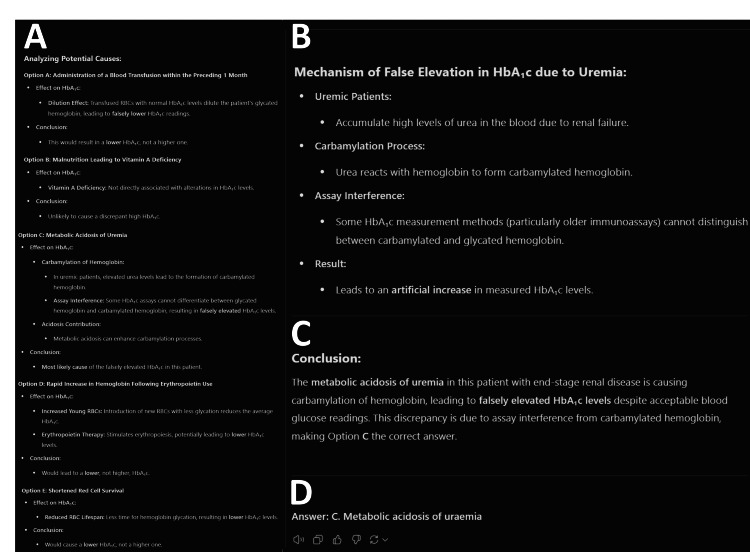
Demonstration of ChatGPT-o1 Preview breakdown and rationalization of each multiple-choice option Figure 4A illustrates how each option was evaluated. Figure 4B shows the possible mechanisms for haemoglobin A1c (HbA1c) elevation relevant to this scenario. Figure 4C shows the conclusion. Figure 4D provides the final recommended answer. Screenshot taken from a ChatGPT chat transcript (ChatGPT; OpenAI, San Francisco, CA, USA).

All LLM responses highlighted the most appropriate multiple-choice option, followed by their logical reasoning using the information provided. The level of detail varied between questions and models, with outputs ranging from 71 to 543 words in length. ChatGPT-o1 Preview generally provided the most reasoning and detail in its responses (Figures 3, 4). FKGL scores for the LLMs ranged between 11 and 14, and FRES ranged between 14 and 31. Three questions included a photograph, and none of the LLMs answered all three correctly. No obvious AI hallucinations were noted in this study.

Performance by LLM and question theme

LLMs differed significantly in exam performance (p<0.001). ChatGPT-o1 Preview provided both the most accurate and the longest responses. Statistically significant differences were present between LLMs for FRES (p<0.001) and FKGL (p=0.004), but not word count (p=0.633). A breakdown of the readability metrics is shown in Table 1.

**Table 1:** Breakdown of median (IQR) readability metrics for each model ChatGPT-o1 Preview (OpenAI, USA), ChatGPT-4o (OpenAI, USA), Gemini (Google, USA), Claude-3.5 Sonnet (Anthropic, USA), Copilot (Microsoft, USA), Perplexity AI (Perplexity, USA), and Meta AI (Meta, USA). FRES: Flesch Reading Ease; FKG: Flesch Kincaid Grade Level; IQR: Interquartile Range

Metric	Claude 3.5-Sonnet	Copilot	Gemini	GPT-o1 Preview	GPT-4o	Meta AI	Perplexity
Word Count	314 (259-342)	59 (47-95)	255 (217-290)	544 (462-608)	309 (265-344)	150 (137-160)	234 (206-262)
FRES	17.8 (16-36)	26.9 (17-33)	29.3 (23-37)	17.8 (9-25)	27.3 (21-33)	12.1 (6-20)	21.9 (14-29)
FKGL	14.4 (11-15)	13.1 (12-15)	12.7 (12-14)	14.4 (13-15)	13.2 (12-14)	14.2 (13-16)	14.7 (14-16)

Twenty-six questions were diabetes-focused and had poorer performance than endocrinology-focused questions (51% vs 60%; p=0.041). A larger proportion of endocrinology-focused questions included reference ranges (82% vs 62%).

**Figure 5:**
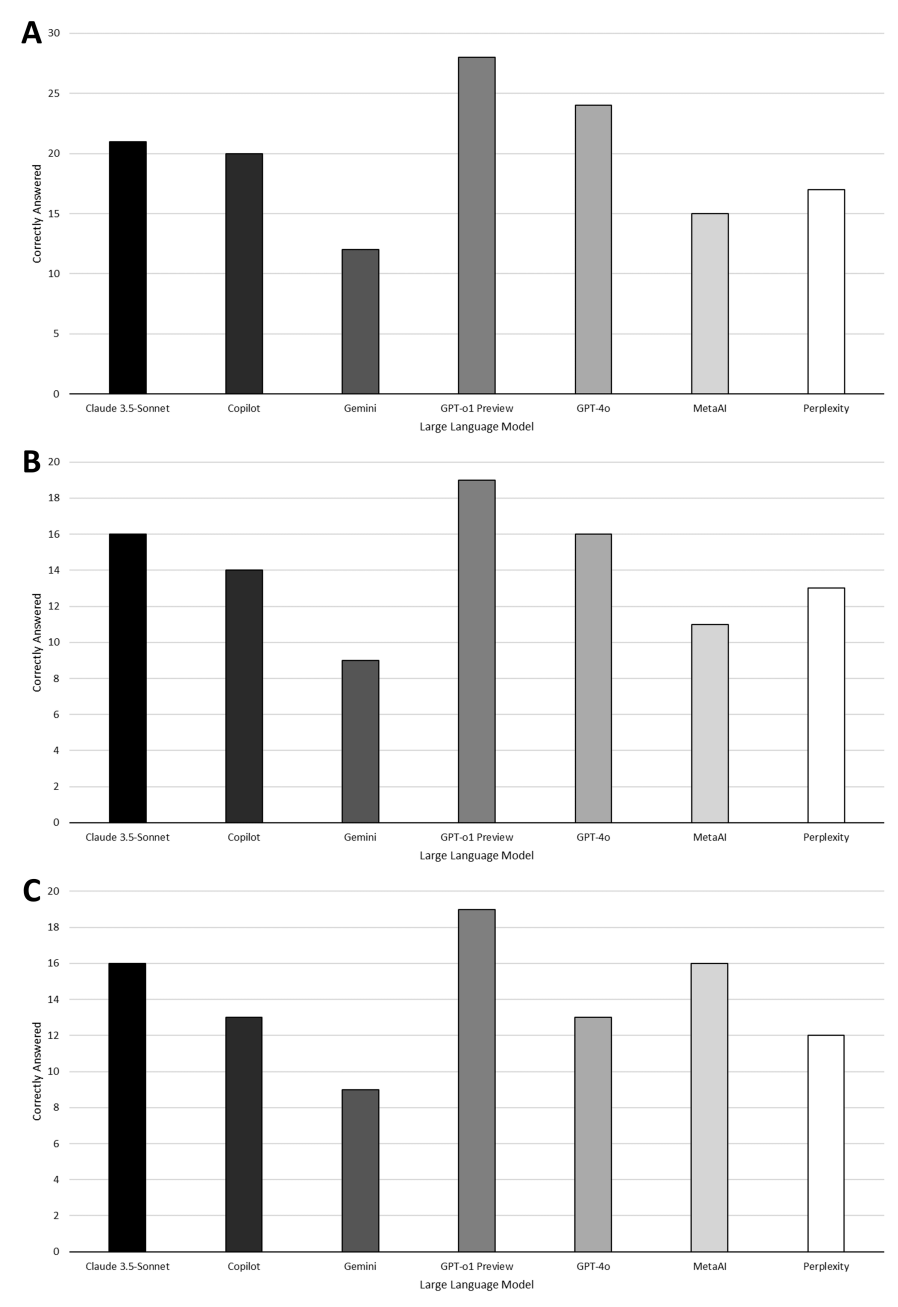
Performance of large language models by question theme Performance on management (Figure 5A; N=37), diagnosis/investigation (Figure 5B; N=24), and physiology & anatomy (Figure 5C; N=21) are shown. ChatGPT-o1 Preview (OpenAI, USA), ChatGPT-4o (OpenAI, USA), Gemini (Google, USA), Claude-3.5 Sonnet (Anthropic, USA), Copilot (Microsoft, USA), Perplexity AI (Perplexity, USA), and Meta AI (Meta, USA).

The performance by question theme (management, diagnosis/investigation, or physiology/anatomy) is shown in Figure 5. The overall performance of LLMs also differed significantly between themes (p=0.011), with better performance in management and physiology/anatomy-themed questions.

Performance by use of reference ranges in question

Sixty-three of the 83 questions included reference ranges. The overall performance of LLMs differed significantly, where questions without reference ranges were more likely to be answered correctly (71% vs 53%; p<0.001).

## Discussion

This study represents one of the most comprehensive assessments and comparisons of the medical knowledge and literacy of LLM outputs to date. With the rapidly growing influence of LLMs within healthcare and medical education, it is crucial to understand the quality and reliability of these resources. With the existence of phenomena causing errors, such as AI hallucination, the ability of these tools should be formally assessed in order to understand how they can be used safely.

Overall performance

The LLMs assessed in this study generally performed poorly, with only ChatGPT-o1 Preview achieving a clear pass in the examination. To date, few studies have evaluated LLMs in the context of diabetes and endocrinology. A 2023 study tested previous iterations of Gemini (Google Bard) and ChatGPT using a curated set of multiple-choice questions, in which these models only achieved scores of 49% and 52% respectively [7]. Both models failed to meet the pre-determined pass mark of 60% and showed that, while they could be applied to attempt these challenges, there was still a noticeable gap in the knowledge or critical thinking ability of these LLMs. These issues appear to apply to most commercially available models and raise questions, as they contrast with studies in other disciplines.

The currently available literature includes studies within other medical and surgical fields, which show variable performance [4,6,14]. Studies evaluating the performance of LLMs in the US Medical Licensing Examination (USMLE) have correlated their performance to the level of third-year medical students and first-year resident doctors [2]. Other, UK-based studies have also shown performance to an appropriate level for the respective examinations [4,15-17]. In general, the greatest concern lies in factually inaccurate or poor clinical advice, as these tools may be used by both clinicians and lay persons. This concern is the greatest for Google’s Gemini, which performed the worst out of the commercially available LLMs, echoing our previous findings in the dermatology SCE [6]. However, with the growing popularity and accessibility of other competing models, such as Meta and Claude, the limited evidence around LLMs and specialty medical knowledge calls for further assessment [7].

Similar to findings by Meo et al., within the few studies that compared LLMs, ChatGPT and Claude have been shown to outperform Google Gemini’s predecessor (Bard) [7]. This highlights the strength of this study as it offers a direct comparison of performance between numerous mainstream LLMs.

Another important aspect is to understand whether the type of question may affect performance. The SCE questions can be broadly categorized into either diagnosis/investigation, management, or physiology/anatomy. The performance of each LLM relative to the others was similar between question themes, but overall, they performed best within physiology/anatomy questions. This could indicate the LLMs’ strengths in ‘factual recall’ as opposed to the greater level of logical and critical thinking required by questions involving clinical scenarios. This could be supported by the fact that ChatGPT-o1 Preview excelled across all question types, as it boasts improved capability in rationalization and critical thinking [18].

Reasoning models

ChatGPT-o1 Preview is an example of a reasoning model, an LLM that can solve more complex problems that require intermediate steps. Examples of non-reasoning models include ChatGPT-4o, Claude 3.5 Sonnet, and Gemini. OpenAI's ChatGPT-o1 Preview claims to more effectively tackle complex reasoning tasks as compared to its predecessor, ChatGPT-4o. ChatGPT-o1 Preview achieves this by spending more time "thinking" to break down problems into intermediate steps and assess multiple options before reaching a conclusion [18]. Answering medical questions, like those encountered in the diabetes and endocrinology SCE, often requires multi-step thinking rather than simple factual recall. For example, a question about the management of a condition may require the candidate to first infer the diagnosis from the given case vignette, then to apply their knowledge of management principles.

The better performance of reasoning models is likely attributable to their iterative processes. However, while reasoning models can provide more nuanced responses, they tend to be slower and require more computational power [19]. Additionally, given the increased reliance on previous outputs, they can also be more prone to AI hallucination and overconfidence [20]. Therefore, in their current form, they are not the most appropriate LLMs for simple knowledge-based or summarization tasks. They are most useful at solving complex problems systematically and can benefit medical learners through a detailed breakdown of reasoning steps. ChatGPT-o1 Preview’s optimistic performance metrics in reasoning tasks have since been followed with the rapid development of further models that rely on ‘chain of thought’ processes, and they continue to push the boundaries of LLM capabilities [21,22].

Other models

The architecture of LLMs also greatly influences outputs and performance. As this study tested the most well-known models, the products selected were designed for general use, which will vastly differ in performance from those trained specifically for healthcare. Additionally, whilst ChatGPT, Gemini, and Claude were developed for general use, Microsoft’s Copilot was designed to synergize with other Microsoft applications. These different goals likely influenced the underlying architecture of these models, which could contribute to the differences in accuracy between models: ChatGPT and Copilot use the Generative Pretrained Transformer whereas Gemini uses the Language Model for Dialogue Application (LaMDA; Google, Mountain View, CA, USA) and Pathways Language Model (PaLM2; Google, Mountain View, CA, USA) [23].

Another low-scoring model was Perplexity, which was designed as an AI-assisted ‘answer engine’ to help answer users’ queries - it may have performed sub-optimally in this study as it strayed from its original purpose [24]. Finally, LLM performance is affected by the dataset used to train and validate the model. Inherent bias in the data itself, as well as the training supervision, will greatly affect how LLMs draw conclusions between data points, and hence how they perform in answering SCE questions.

Language

The difference in quality of language produced by the LLM models was also statistically significant, albeit the range in quality of language was limited to FKGL scores of 13 to 15 (roughly equivalent to the reading level of an American college student). This demonstrates the overall ability of LLMs to generate responses appropriate to the literacy level of the user posing prompts. The outputs in this study were likely generated using the academic medical literature from which the LLMs were recalling answers, hence resulting in text that reflected higher than average literacy levels [11,25]. ChatGPT-o1 Preview delivered both the best performance in the SCE and the highest word count, which was likely due to the inclusion of the ‘thought process’ of the reasoning model (Figure 3). This makes ChatGPT-o1 Preview the most likely to be useful for learners as it provides the most detailed explanation on the logical progression between facts, information in the question stem, and its conclusion (Figure 4).

The findings in this study likely also apply to content in non-English languages. This is rarely explored, but the LLMs used in this study have generally shown reduced performance when used within the medical field in other languages, including German, Japanese, and Polish [26-28]. While performance in other languages did not fall within the scope of this study, these LLMs are likely to be inadequate in responding to non-English medical specialty-related prompts. This may be due to the relative lack of non-English medical literature, with 96% of PubMed-indexed articles in 2015 being in English [29]. This likely reduces the ability of LLMs to form meaningful associations between data points and parameters. Nonetheless, further studies evaluating this may shed more light on the role of LLMs for medical professionals in non-English settings.

Prompting

Prompt engineering is an increasingly important consideration for LLM users, as inputting specific requests can help LLMs to tailor their output and adhere to certain formats. Specific prompts were not included in this study as unprompted use provides insight into the baseline performance of LLMs. This study assumed that normal users do not pre-prompt and acknowledges that the wide variation in pre-prompt wording can lead to drastically different outcomes [11].

One concern explored during this study was whether the format of the questions themselves could potentially affect LLM performance [30]. A subgroup analysis compared performance in questions with and without reference ranges. Overall, LLMs appeared to answer questions more accurately when not presented with reference ranges (p<0.001). This was likely multifactorial, as the nature of questions may have differed between those with and without reference ranges, such as testing knowledge from different topic areas. While there was no obvious AI hallucination in this study, the inclusion of reference ranges itself could have independently ‘confused’ the LLMs. Therefore, this supports the notion that providing excess information can sometimes be counterintuitive and lead to poorer LLM performance.

Implications

LLMs represent the latest technological advancement with the potential to improve healthcare delivery and education [1]. The comprehensive datasets used by LLM trainers allow models to develop relationships between countless parameters and variables. While LLMs can respond to structured scenarios and multiple-choice questions, their performance in this study showcased their strengths and weaknesses. With increasing computing power and more complex AI architectures, the capabilities of these models will likely continue to improve, allowing them to become better tools for medical education. However, it is also important for users to be aware of the limitations of AI, such as over-fitting and biases, as models may not always perform better with larger datasets. 

The findings from this study have shown that there are significant differences in the performance of LLMs, likely secondary to the architecture and training data sets of the models, amongst other factors. We hope that these findings help clinicians to make more informed choices when considering the use of LLMs as educational aids. In particular, the choice of LLM is most important if used for questions requiring a greater level of critical thinking, where the newer, reasoning models such as ChatGPT-o1 Preview have higher accuracy and may provide more detailed explanations to learn from. 

The strength of this study is the multi-dimensional assessment of a wide variety of commercially available LLMs. This study evaluated their performance in the field of diabetes and endocrinology by testing the LLMs on a small sample of quality-assured examination questions with validated answers. Nonetheless, further studies should be conducted to understand the real-world implications of LLMs on medical knowledge and clinical performance, as well as how they are being utilized by healthcare professionals. Research in this field should continue to explore the uses and limitations of models, keeping pace with the rapid development of new models as they become available. LLMs, and especially reasoning models, hold potential for improving postgraduate medical education through enabling a more personalized learning experience for trainees [8].

## Conclusions

This comprehensive study highlights the strengths and limitations of modern LLMs using a specialist diabetes and endocrinology examination. While the performance was generally poor amongst the mainstream models, ChatGPT-o1 Preview, a reasoning model, showed significantly better performance than other models and achieved the historical passing grade. These newer iterations of LLMs may be useful adjuncts to traditional clinical learning. Users should continue to explore the benefits of LLMs in medical education and how this translates to clinical practice, but should be aware that their performance is far from perfect and can often be inaccurate.
